# Formulation-Driven Rational Architectonic Design of Pomelo (*Citrus maxima*) Peel-Derived Lignocellulosic Fibre-Enriched Chewable Tablets: Spectroscopic and Morphological Characterization of Structure–Function Relationships

**DOI:** 10.3390/foods15111898

**Published:** 2026-05-28

**Authors:** Saadullah Arslan Ahmad, Lufeng Wang, Atif Arshad

**Affiliations:** 1 College of Food Science and Technology, Huazhong Agricultural University, Wuhan 430070, China; saadullahahmad5380@gmail.com; 2 Anhui Engineering Research Center for High Value Utilization of Characteristic Agricultural Products, School of Food and Nutrition, Anhui Agricultural University, Hefei 230036, China; atifarshad0000@gmail.com

**Keywords:** chewable tablets, pomelo peel fibre, formulation and characterisation, physicochemical properties, value-added product, dietary supplement

## Abstract

Dietary fibre deficiency remains a global nutritional concern, and the development of fibre-enriched functional foods is increasingly important. Fruit processing by-products, such as pomelo (*Citrus maxima*) peel, represent a sustainable source of dietary fibre with potential techno-functional applications. In this study, a fibre-enriched chewable tablet was developed using pomelo (*Citrus maxima*) peel fibre (CPF) as a functional ingredient and structural matrix. The formulation was prepared through direct compression and systematically evaluated in terms of powder flow behaviour, structural characteristics (FTIR, XRD, and SEM), hydration-related functionality, and tablet quality attributes. The CPF-based powder blend exhibited good flowability and compressibility, with an angle of repose of 26.62°, Carr’s index of 8.9%, and a Hausner ratio of 1.22, indicating suitability for tablet processing. Structural characterisation demonstrated the formation of a semi-crystalline matrix with a porous fibrous morphology. CPF incorporation significantly improved water-holding capacity (9.84 g/g) and oil-holding capacity (2.97 g/g), indicating enhanced hydration functionality. The developed tablets exhibited uniform weight (560 ± 9.5 mg), consistent dimensions, and acceptable disintegration behaviour (168 ± 15 s), demonstrating satisfactory physical quality. Overall, these findings suggest that pomelo peel fibre can be effectively utilised in chewable delivery systems, providing a feasible strategy for the valorisation of citrus by-products in functional food applications.

## 1. Introduction

Recently, obesity and overweight have become a critical public health concern in the global scene. Dietary plans that evoke a sense of fullness have also received significant interest as possible regulators of energy consumption and body mass. Interestingly, the increase in dietary fibre consumption has been reported to help in weight control by suppressing hunger signals and extending the satiety after eating [1,2].

Dietary fibre (DF) is a complicated category of non-digestible vegetable polysaccharides, which supplies insignificant caloric energy to the human physique [3]. DF is typically split into two major groups based on solubility in aqueous solution: insoluble dietary fibre (IDF) and soluble dietary fibre (SDF) [4]. Adequate intake of dietary fibre has been associated with reduced risks of obesity, cardiovascular diseases, and type 2 diabetes, and is therefore widely recommended in dietary guidelines [5]. The World Health Organisation (WHO) indicates that adults need to consume a minimum of 25 g of fibre per day in order to have proper physiological and metabolic health [6]. However, epidemiological statistics showed that almost 90 percent of the American population does not meet these guidelines, with an average daily fibre intake of 15 g/day. To counter this widespread fibre gap, numerous consumers have been turning to single-source fibre supplements. Nevertheless, these solitary fibre fractions do not contain complex physicochemical characteristics and synergistic bioactive elements of whole foods, and thus cannot be assumed to have the same physiological effects as whole foods [7]. Dietary fibre mainly consists of cellulose, hemicellulose, lignin, pectin, and related structural polysaccharides, which contribute to their hydration behaviour, compressibility, and structural functionality in formulated systems [8].

In this context, the utilisation of fruit processing by-products, such as pomelo (*Citrus maxima*) peel, represents a promising and sustainable source of dietary fibre with potential techno-functional applications. Citrus processing residues are among the most abundant lignocellulosic agricultural by-products generated worldwide, with citrus peels accounting for approximately 40–60% of the total fruit mass during industrial processing [9]. These by-products are rich in cellulose, hemicellulose, pectin, flavonoids, and dietary fibre fractions, making them promising materials for value-added food applications [10]. The stability of bioactive constituents and the inherent physico-mechanical characteristics of pomelo peel fibre present a major formulation challenge. The coarse and rigid nature of the peel may give the structure an unwanted mouthfeel, thus constraining its use directly within the functional food systems [11]. Therefore, to create an acceptable chewable dosage form, accurate matrix engineering is required. This includes the rational choice of excipients and optimisation of formulation factors to control textural properties, providing a cohesive, smooth, palatable product without compromising the structural integrity and functional performance of the fibre [12].

The possibility of delivering multiple functional attributes in a single convenient chewable formulation has recently gained increasing interest because of its technological features, the convenience of the formulation, and its consumer acceptance [13]. Despite these benefits, the use of citrus peel-based lignocellulosic fibre in chewable tablet formulations has not been fully investigated, especially regarding its structural interactions in direct-compression matrices, compressibility, and its physicochemical functionality. Most of the previous research related to pomelo peel fibre has been focused on its incorporation into bakery products, beverages, edible films, and traditional functional foods, with only a few studies on the use of pomelo peel fibre in solid chewable delivery systems [14]. Furthermore, the multiscale characterisation of citrus fibre-based chewable matrices using spectroscopic, crystallographic, morphological, and thermal analysis is very limited in the literature. Thus, the present study focused on the development of pomelo (*Citrus maxima*) peel-based fibre-enriched chewable tablet by direct compression. The physicochemical, hydration, structural, and morphological properties of the prepared tablets were systematically evaluated. The study also investigated the functional properties of the citrus peel fibre by comparing it to a maltitol control formulation. FTIR, XRD, SEM, and particle size evaluation were used to explain the formulation behaviour and structural properties of the prepared chewable matrices.

Therefore, the objective of this study was to develop and evaluate a fibre-enriched chewable tablet using pomelo peel fibre as a functional ingredient. The formulation was prepared using a direct compression approach and systematically characterised in terms of micromeritic properties, structural attributes, physicochemical functionality, and tablet quality characteristics. Comparative evaluation with a maltitol-based control formulation was also carried out to investigate the techno-functional contribution of pomelo peel-derived fibre within the chewable tablet matrix. In addition, advanced characterisation techniques, including Fourier-transform infrared spectroscopy (FTIR), X-ray diffraction (XRD), scanning electron microscopy (SEM), and particle size analysis, were employed to investigate the structural organisation and formulation behaviour of the developed chewable systems. This work aimed to investigate the feasibility of utilising pomelo peel fibre in chewable tablet formulations through comprehensive physicochemical, structural, and technological characterisation and to provide a basis for its application in functional food development.

## 2. Materials and Methods

### 2.1. Materials and Reagents

The high-fibre ingredient was the Citrus peel fibre (CPF) of Pomelo (*Citrus maxima*), which was obtained and utilised in the Huazhong Agricultural University, Hubei, Wuhan laboratory. Fresh pomelo peels were thoroughly washed to remove surface impurities, dried under controlled laboratory conditions, ground into powder form, and passed through a 50-mesh sieve to obtain a relatively uniform particle size prior to formulation preparation. The prepared CPF powder was stored in airtight containers at room temperature until further use. Other excipients included pharmaceutical-grade excipients such as microcrystalline cellulose (MCC, filler/binder), maltitol (powdered sweet diluent), citric acid (acidulant), orange powder (flavouring/colouring agent), and stevia (sweetener) to improve palatability. All chemicals and reagents used in the study were of analytical grade. These were provided by the Sinopharm Chemical Reagent Co., Ltd. (Shanghai, China).

### 2.2. Preparation, Optimisation, and Compression of CPF-Enriched Chewable Tablet Formulations

The pomelo peel fibre (CPF) chewable tablets were optimised by the direct compression method with the composition of excipients and compression parameters, and the tablets were prepared. All excipients were carefully weighed, as per the optimised formulation, as given in Table 1. To achieve a uniform particle size distribution and to reduce any agglomeration of powder materials, they were passed through a 50-mesh sieve before blending. The formulation process was performed under controlled laboratory conditions at ambient temperature (25 ± 2 °C) and relative humidity (RH) of about 45–55% [15]. The powder blending process was carried out in three steps based on the function of excipients. CPF (or maltitol in the control formulation), oligosaccharide, orange powder, and microcrystalline cellulose (MCC) were thoroughly blended together to ensure the uniform distribution of the major components of the matrix by using a mortar and pestle for about 10 min. In the secondary blending step, citric acid and stevia were added to the pre-blended formulation and blended for another 5–10 min to blend them uniformly and distribute their flavours throughout the formulation. During the final step of lubrication, magnesium stearate was incorporated and very gently blended for about 3 min to prevent over-lubrication and maintain the compressibility properties. All the final powder blends were subjected to the micromeritic property test before the compression [16].

The compression of tablets was carried out by a single-punch eccentric tablet press using the direct compression method. Flat-faced cylindrical punches with diameters of 10 mm were used to prepare the chewable tablets. To obtain tablets with satisfactory dimensional uniformity, disintegration pattern, and structural integrity, preliminary optimisation trials were carried out with varying compression pressures. The optimisation process was conducted through preliminary single-factor trials in which powder flowability, compressibility, tablet integrity, dimensional uniformity, and disintegration behaviour were systematically evaluated. Formulations showing poor flow properties, high friability, capping, lamination, and non-uniform tablet size were eliminated during the optimisation process. Based on these trials, it was determined that a moderate compression force range of about 5–7 kN was optimal to ensure that the tablets were not too hard to compress yet had satisfactory mechanical strength for tablet handling and storage. All formulation batches were made up of about 30 tablets compressed under the same conditions to ensure batch-to-batch reproducibility. A control formulation was also prepared following the same formulation and processing conditions, but with complete replacement of CPF on a weight-equivalent basis using maltitol. Comparative evaluation between CPF and control formulations was then conducted to explore the technological contribution of pomelo peel fibre within the chewable tablet matrix system. The optimised formulation obtained from preliminary trials was used for further characterisation.

### 2.3. Powder Micromeritic Characterisation

In physicochemical testing, it is necessary to establish the physical properties of the mixed powder excipients to determine adherence to the pharmacopoeial standards. Moreover, the analysis of the basic physical constants was conducted within the methodological guidelines [17].

#### 2.3.1. Angle of Repose

The fixed funnel method was used for measurements [18]. The powdered sample was left to pass through a funnel that was attached to a burette stand at a constant height of about 4 cm. A sheet of graph paper was put at the bottom of the funnel to collect the powder to help measure the conical heap formed accurately. Measurements were taken of the height (h) and radius (r) of the powder heap, and the angle of repose (θ) was determined using the equation below:

(1) tanθ=h/r

#### 2.3.2. Determination of Bulk Density

A 5.0 g portion of the powdered sample was weighed and transferred into a clean graduated cylinder with the aid of a funnel. After gently tapping the cylinder to allow the powder to settle, the observed volume was recorded [17]. Measurement of the volume occupied by the powder was taken, and bulk density was calculated by the following formula:

(2) $$Bulk\;Density=\frac{Mass\;of\;powder}{Bulk\;volume}$$

#### 2.3.3. Determination of Tapped Density

The density of the tapped sample was calculated by weighing the powdered sample to the nearest gram (10 g) and placing it in a clean graduated cylinder. Mechanical tapping of the cylinder was done with a specified number of strokes to ensure the packing of the particles, and the volume of the resultant was noted. The tapping procedure was continued for about 10–15 min or until no additional decrease in volume was noticed, which indicates a state of compaction equilibrium. The tapped density (ρn) was determined as the ratio of the mass of the powder to the final tapped volume (g/cm^3^) [1,19].

(3) $$Tapped\;Density=\frac{Mass\;of\;powder}{Tapped\;volume}$$

#### 2.3.4. Hausner’s Ratio and Carr’s Index

The ratio of tapped density to bulk density was computed by Hausner to give a quantitative result of the flowability and compressibility properties of the powder [1].

(4) $$Hausner’s\;Ratio=\frac{Tapped\;Density}{Bulk\;Density}$$

Carr’s index determines the compressibility of the powder. Based on the apparent bulk density and tapped density, the percentage compressibility of the powder can be determined. A Carr’s index > 25 is considered to be an indication of poor flowability, and below 15 of good flowability. Carr’s index (Compressibility Index):

(5) $$Carr’s\;Index\left(\%\right)=\frac{Tapped\;Density-Bulk\;Density}{Tapped\;Density}\times 100$$

This index measures the compressibility and flow behaviour of powders.

### 2.4. Structural Analysis

#### 2.4.1. Fourier Transform Infrared Spectroscopy (FTIR)

The Fourier Transform Infrared (FTIR) Spectroscopy analysis was performed as per the procedure followed: The samples were dried, and then a 1:100 (*w*/*w*) mixture of spectroscopic-grade potassium bromide (KBr) and the sample was ground uniformly into a fine powder. The mixture obtained was compressed into a stainless-steel die in a hydraulic press to form transparent pellets [20]. Pure KBr was scanned before the measurement of the sample to get a background spectrum to correct the sample. A Nexus 470 FTIR spectrometer (Nicolet Instrument Corporation, Madison, WI, USA) was used to record the infrared spectra over the wavelength range of 400–4000 cm^−1^, with a resolution of 4 cm^−1^, with 32 cumulative scans per sample.

#### 2.4.2. X-Ray Diffractometric Analysis (XRD)

An X-ray diffractometer (D8 Advance, Bruker AXS GmbH, Karlsruhe, Germany) was used to obtain the crystallinity profile of the samples. The calculation was done using a Cu-K alpha radiation source (λ = 0.154 nm) at an accelerating voltage of 36 kV and 20 mA current. The diffraction patterns were recorded in the 2θ range of 5°−55°, with a scanning speed of 10° min^−1^, to record the typical peaks of crystalline and amorphous regions [21]. The crystallinity index (CI) of each sample was calculated following the procedure described by Xiao et al. [21], which gives a quantitative measure of the relative level of structural order in the sample matrix.

#### 2.4.3. Scanning Electron Microscopy (SEM)

A scanning electron microscope (JSM-6390, JEOL Ltd., Tokyo, Japan) was used to study the microstructure of fibre samples. The samples were gold-sputtered before imaging, and were observed at an acceleration voltage of 10 kV, and micrographs were recorded at magnifications of 200×, 1000×, and 2000×.

### 2.5. Physicochemical Properties

#### 2.5.1. Water-Holding Capacity (WHC)

The determination of water-holding capacity (WHC) of samples was also based on the procedure provided as follows: The sample (W1) was weighed exactly 0.10 g and added to a centrifuge tube (W2), which was pre-weighed, and then 15 mL of distilled water was added. The suspension was completely mixed to provide homogeneous dispersion and then left to stand at 25 °C after 24 h to gain full hydration [22].

The mixture was incubated and centrifuged at 5000 rpm for a few minutes, and the supernatant was decanted carefully. The weight of the centrifuge tube and the wet residue (W3) was then measured together (precisely). The WHC calculation was based on the following Equation (6).

(6) $$WHC(g/g)=\frac{W3-W2-W1}{W1}$$

#### 2.5.2. Oil-Holding Capacity (OHC)

The oil-holding capacity (OHC) of the samples was measured based on the approach described in the article by [23], with minor changes. The sample (W1) was weighed accurately and put in a pre-weighed centrifuge tube (W2), and 15 mL of corn oil was added. The mixture was thoroughly mixed for 1 h in order to make sure that the sample and oil phase had fully dispersed and interacted. Then the tubes were centrifuged at a speed of 5000 rpm for 15 min. The centrifugation was followed by carefully decanting the supernatant oil and wiping the inside of the tube to remove any residual oil. The total weight of the tube and the residue that remained (W3) was measured. The oil-holding capacity was calculated using Equation (7):

(7) $$OHC(g/g)=\frac{W3-W2-W1}{W1}$$

#### 2.5.3. Particle Size Distribution

The size distribution of the powder samples, CPF and maltitol, was measured using a laser diffraction particle size analyser (Mastersizer 2000, Malvern Instrument Ltd., Worcestershire, UK) in the procedure as follows: The samples were dispersed in distilled water to form a 0.2% (*w*/*v*) suspension before the measurement, and the suspension was uniformly dispersed [24]. In the analysis, the refractive indices of powder samples and aqueous phase were adjusted to 1.48 and 1.33, respectively. The size of the particles was reported in terms of the volume-weighted mean diameter (D_4,3_ µm).

### 2.6. Preparation and Evaluation of Chewable Tablets

#### 2.6.1. Tablet Compression, Physical Appearance, and Shape Analysis of Chewable Tablets

The blended mixture was then compressed into tablets using different compression forces, and a single-punch machine was used to create tablets that were nearly identical in size, ranging from 10 mm to 12 mm, with flat punches.

The visual appearance and geometry of chewable tablets were assessed under adequate laboratory lighting conditions to examine the colour, surface appearance, and integrity of the product. The shape (circular and flat) was visually verified, and the size uniformity (tablet diameter and thickness) was determined using a calibrated digital Vernier calliper or micrometre. Several tablets (*n* ≥ 3) were randomly selected from each batch for representativeness to assess uniformity and compaction characteristics, as tablet thickness and diameter relate to die filling, compression pressure, and tablet uniformity, respectively. Furthermore, visual and dimensional characteristics are known to be critical attributes for product acceptability, processability, and performance of solid drug products.

#### 2.6.2. Weight Variation

Twenty tablets of each batch of formulation were randomly picked and weighed separately and in batches using a calibrated digital analytical balance to determine accuracy. The average weight of the tablet was calculated by dividing the total weight by the number of tablets. The weight of the individual tablets was then compared to the calculated mean to establish consistency in weight. It was calculated and assessed as a percentage deviation of the weights of individual tablets compared with the average value of the weight of the tablet as a percentage of the permissible pharmacopoeial variation [25].

#### 2.6.3. Thickness and Diameter

Tablet thickness and diameter are crucial quality control parameters because they directly indicate the behaviour of compression, consistency of die fill, and the overall consistency of the dosage form. The difference in these dimensions may affect the mechanical strength, porosity, and disintegration behaviour of the tablets. Digital callipers and micrometres are commonly used as standard micrometric methods of achieving this aim, owing to their accuracy and repeatability in pharmaceutical analysis [26]. In addition, dimensional aspects like thickness and diameter are needed to determine the volume and density of the tablet, which is strongly linked to the compaction properties and internal fabric of the formulation.

The thickness and diameter of the chewable tablets were measured using a calibrated digital Vernier calliper (±0.01 mm accuracy). Each batch of formulation was randomly chosen and measured (*n* = 3), with a minimum of three tablets per batch. The diameter of the tablet was taken as the widest plane, and the thickness was taken as the central height of the tablet. The measurements were conducted in controlled laboratory conditions to reduce variability caused by environmental factors. Mean values and SD were determined to evaluate the uniformity and reproducibility of the tablet formulations in terms of dimensions [27].

#### 2.6.4. Disintegration Test

The disintegration test was used to determine cooking time under controlled experimental conditions to disintegrate a tablet sample into fine particles that could pass through a 10-mesh sieve. The evaluation was done through a disintegration apparatus with a basket rack that was filled with six cylindrical plastic tubes, open at both ends, and lined at the lower part with a 10-mesh screen. The basket assembly was dipped in a beaker filled with a suitable testing medium kept at 37 ± 0.5 °C. In the case of uncoated compressed tablets, the medium of disintegration was the distilled water kept at the same temperature. If one or two tablets failed to disintegrate after the designated time, the test was repeated with twelve tablets. The allowable time of disintegration to be used to comply was calculated as per the requirements indicated in the corresponding pharmacopeial monographs, which guaranteed the quality and consistency of goods [28].

### 2.7. Statistical Analysis

All experiments were conducted in triplicate using independent experimental replicates. The results obtained were presented as mean values with standard deviation (SD) and represented in the form of graphs with the help of OriginPro 2022. Statistical tests were performed using IBM SPSS Statistics 27. One-way analysis of variance (ANOVA) was used to test differences between sample means, with Duncan’s multiple range test used to determine significant differences between groups. The level of statistical significance was taken at *p* < 0.05.

## 3. Results and Discussion

### 3.1. Micromeritic Properties of Powder Blends

#### 3.1.1. Analysis of the Angle of Repose

**Figure 1:**
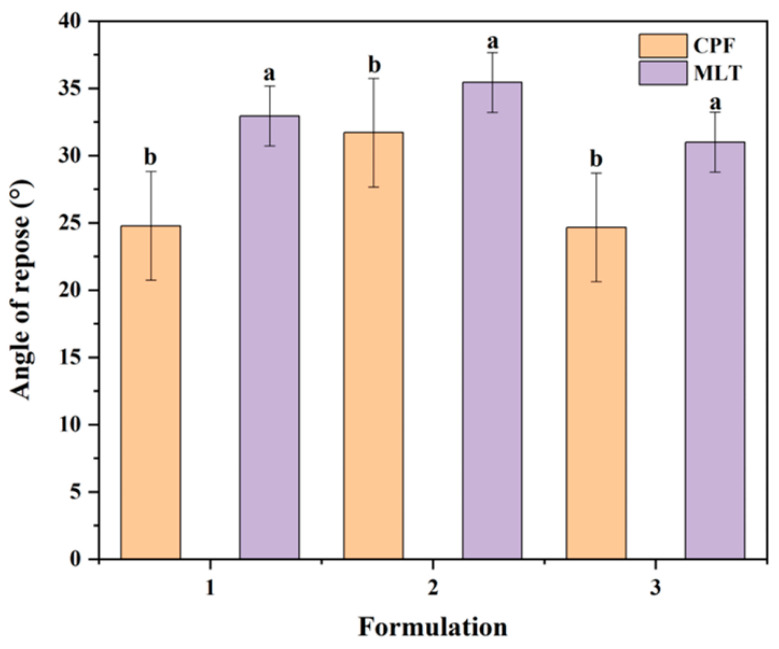
Angle of repose (°) of CPF and maltitol (MLT) powder formulations. Different lowercase letters represent significant differences.

The physicochemical characteristics of the individual bioactive powders and their formulated blends were carefully studied to evaluate their quality, compositional integrity, and adherence to standard parameters (Table 2 and Figure 1). An optimised angle of repose for the citrus peel fibre (CPF) powder blend was determined to be 26.62 ± 3.53°, which represented excellent flow properties. Angle of repose is an established parameter to measure the flowability of powders, which is a measure of the degree of interparticle friction and cohesion within the system. Reduced values denote reduced resistance among particles, which results in better dynamics of flow under the pressure of gravity. Under usual pharmaceutical grading, the angle of repose of 25–30° indicates good flowability, which is highly beneficial in direct compression operations. The value obtained shows that the refined formula has minimal interparticle friction and cohesive forces, and thus is easy to transfer particles in the processing [17].

The excellent flow characteristics observed in this study can be attributed to the optimised excipient mixture that was described in Section 3.1. Microcrystalline cellulose was used, and this greatly increased flowability as the powder packing properties were improved and particle friction was reduced. The availability of oligosaccharides and controlled moisture content increased the particle contact and reduced the aggregation. The findings on the angle of repose are consistent with the pre-formulation technique, justifying that the incremental blending process was effective in reducing segregation and increasing the homogeneity of the particles. This is particularly important in recipes containing dietary fibres such as citrus peel fibre, which tend to have poor flow due to their non-uniform shape and fibrous nature.

#### 3.1.2. Bulk Density and Tapped Density Analysis

**Figure 2:**
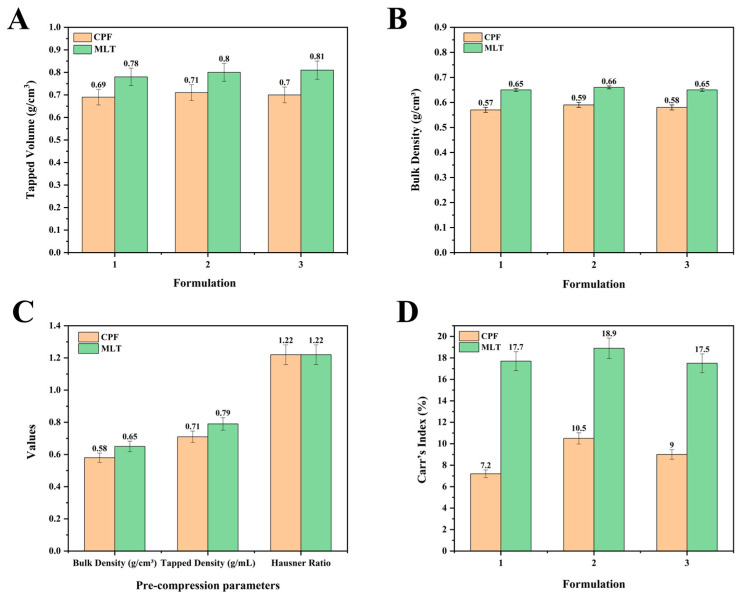
Graphical representation of bulk density (g/cm^3^) (**A**); tapped density (g/cm^3^) (**B**); comparative graphical representation, illustrating the flow and compressibility behaviour of the formulation (**C**) and Carr’s index (**D**).

The optimised citrus peel fibre (CPF) powder blend was found to have a bulk density of 0.58 ± 0.02 g/cm^3^ (Table 3 and Figure 2A), which indicates moderate packing capacity and good flow characteristics. Bulk density is a critical value that shows the spatial structure of particles and the extent of interparticle pores in a powder system. It is influenced by such factors as particle size, morphology, surface texture, and moisture content. The bulk density measured shows that the powder blend has an optimum packing structure, which is important in the uniform filling of the die during the crushing of the tablet [29]. Powders of low bulk density tend to have poor flow due to the increased spaces, and high density could indicate tight packing and reduced compressibility. As a result, the value obtained represents an optimum balance that is suitable for direct compression processes. The results of the bulk density experiment are consistent with those of the angle of repose (26.62°), indicating better flowability. The combination of the two values suggests that the updated formulation exhibits desirable flow properties, which are paramount in attaining equal weight distribution and uniform quality of tablets.

The optimised citrus peel fibre (CPF) powder blend was tapped, and the density of the tap was 0.71 ± 0.03 g/cm^3^ (Table 3 and Figure 2B), which corresponds to the ability of the particles of the powder to rearrange themselves and acquire a more solid packing structure under mechanical tapping. Tapped density is the ratio of the mass of powder to the volume occupied by the powder after a standardised tapping process to reduce interparticle voids and increase the density of packing [17]. It is an important metric in the pharmaceutical formulation because it shows the efficiency in packing and compressibility properties of powder systems. When the tapped density was compared to the bulk density that was determined earlier (0.58 ± 0.02 g/cm^3^), it was revealed that the density increased significantly after tapping. This increase is expected because the bulk density is used to indicate the loose arrangement of the powder particle with significant voids, whereas the tapped density is used to indicate the compaction state achieved by mechanical consolidation [30]. The observed increase of 0.58 to 0.71 g/cm^3^ indicates successful reorganisation of the particles and a reduction in the volume of voids within the powder bed. This behaviour suggests the formulation has a small value of interparticle cohesion and the ability to reform under mechanical stress imposed.

#### 3.1.3. Hausner’s Ratio and Carr’s Index Analysis

The Hausner ratio of the optimised citrus peel fibre (CPF) powder blend using the ratio of tapped density (0.71 g/cm^3^) and bulk density (0.58 g/cm^3^) was calculated as 1.22 (Table 4 and Figure 2C). The Hausner ratio is a commonly used measure of flowability and compressibility of powder systems because it shows the degree of cohesiveness and interparticle friction in the formulation. It gives a quantitative estimate of the behaviour of powder consolidation by calculating based on the difference between tapped packing (tapped density) and loose packing (bulk density). The obtained value of 1.22 indicates that the powder blend is well flowing. Hausner ratios under 1.25 indicate powders that have acceptable to good flow characteristics, and higher values indicate poor flowability due to increased cohesiveness, as per conventional pharmaceutical classification. In this way, the result of the study confirms that the enhanced formulation possesses favourable flow properties suitable for direct compression operations [31]. In general, the Hausner ratio experiment indicates the suitability of the optimised powder blend as a direct compression filler and in the production of chewable tablets by establishing that it is highly flowable and compressible sufficiently. The formulation has a consistent and strong micromeritic profile, which is necessary to produce high-quality tablet formulations, and when the micromeritic profile is measured in conjunction with the values of bulk density, tapped density, and angle of repose.

The citrus peel fibre (CPF) powder blend was optimised and was observed to possess enhanced compressibility and flow properties and a Carr index of 8.9 ± 2.11% (Table 5 and Figure 2D). The index of Carr, often called the compressibility index, is a commonly used index that quantifies the ability of a powder to decrease in volume under the influence of mechanical stress, which is a measure of cohesiveness and the interactions between particles in the system [19]. The index values of Carr below 15% reveal good-to-excellent flow properties, but larger values reveal greater cohesiveness and poor flow behaviour, according to the traditional pharmaceutical classification. The obtained value of less than 10 percent shows that it has a great flowability [32]. The low compressibility index revealed in this study indicates that the resistance to movement of particles and interparticle friction is low, and this allows the powder to move freely and evenly. Furthermore, low compressibility index powders are also more prone to exhibiting uniform tablet hardness, die filling, and reduced weight dispersion, which results in Carr’s index being a predictive variable in tablet manufacturing performance. Consequently, the value obtained proves the suitability of the formulation to make high-quality chewable tablets and justifies its direct compressibility. All of the comparative studies are concluded in Table 6.

### 3.2. Structural Properties

#### 3.2.1. FTIR Analysis

**Figure 3:**
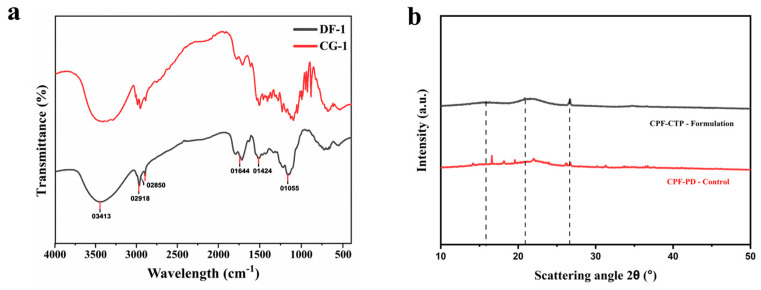
(**a**) FTIR spectra of control and formulated samples, illustrating structural organisation, crystallinity, and functional group interactions within the developed formulation; (**b**) X-ray diffraction patterns.

The infrared spectra of the samples show similar characteristic absorption patterns (Figure 3a), indicating that the structural and chemical compositions of the two samples are relatively similar. The presence of a wide band at 3403 cm^−1^ implies O-H stretching, which is characteristic of hydroxyl-containing groups in cellulose, hemicellulose, lignin, and adsorbed moisture. The strong aliphatic C-H stretching modes (2924 cm^−1^) are found attributed to asymmetrical stretching of CH2/CH3 groups in long hydrocarbon chains. A 1639 cm^−1^ band may be attributed to bound water absorption and aromatic skeletal vibrations associated with lignin components [33]. The highest peak at 1436 cm^−1^ is attributed to CH2/CH3 vibrations related to cellulose and hemicellulosic polysaccharides, the absorption at 1050 cm^−1^ is attributed to C-O stretching vibrations associated with glycosidic linkages and polysaccharide structures, as well as other vibrations in the fingerprint region [34]. Taken together, these spectral characteristics confirm the presence of typical lignocellulosic and polysaccharide-associated functional groups within the pomelo peel fibre matrix.

#### 3.2.2. X-Ray Diffraction

The crystalline structure, crystal type, and degree of crystallinity of cellulose fibre-based and maltitol-based samples, with or without other excipients in the chewable tablet preparations, were analysed by X-ray diffraction (XRD). Figure 3b shows the diffraction profiles of the four samples. The CPF tablets showed a strong and wide diffraction peak at the position of 20–23° (2θ), indicating the presence of an amorphous or semi-crystalline matrix due to the presence of pectin polysaccharides, cellulose, and hemicellulose in the case of citrus peel fibre, which had the typical cellulose crystallographic structure [35]. The similarity of the positions of CPF and MLT and other excipient formulations on the peak positions suggests that the basic crystal structure of maltitol in the formulation did not change fundamentally due to the processing. The characteristic is in line with previous reports that pectin and dietary fibres obtained after the extraction of citrus by-products are amorphous [36]. Conversely, the maltitol-based formulation exhibited very clear, sharp diffraction peaks at the 2θ range of 19–30, and this range of 2θ is typical of a well-defined crystalline structure [37]. The fact that these intense reflections take place proves that even when maltitol is under compression, it still possesses its crystallinity. Conversely, the CPF matrix is mostly disordered due to the difference in molecular organisation between the two excipient systems.

The variation in crystal structure directly relates to the functionality and sensory characteristics of the chewable tablets. The increased amorphous content of the CPF-enriched formulation should enhance compressibility, quicker disintegration, and greater moisture retention, all of which are beneficial characteristics of chewable dosage forms [21]. The strong crystallinity of maltitol, on the other hand, offers hardness and structural rigidity but can lead to slower disintegration and a brittle texture. Other types of amorphous character and enhanced tablet properties have also been observed to be similar to fibre-based formulations with citrus, apple, and other pectic fibres [32]. Overall, the XRD analysis shows that replacing maltitol with CPF will convert the matrix from a crystalline polyol network into a semi-crystalline biopolymeric system. This structural change is likely the basis of the enhanced compressibility, chewability, and the possible functional advantages (e.g., dietary fibre fortification and reduced sugar content) of CPF-fortified chewable tablets. To further confirm the observed reduction in crystallinity, and to explain the interactions between the fibres and excipients that led to the altered microstructure of the tablets, complementary solid-state studies like DSC, FTIR, and SEM were conducted.

#### 3.2.3. Scanning Electron Microscopy (SEM)

**Figure 4:**
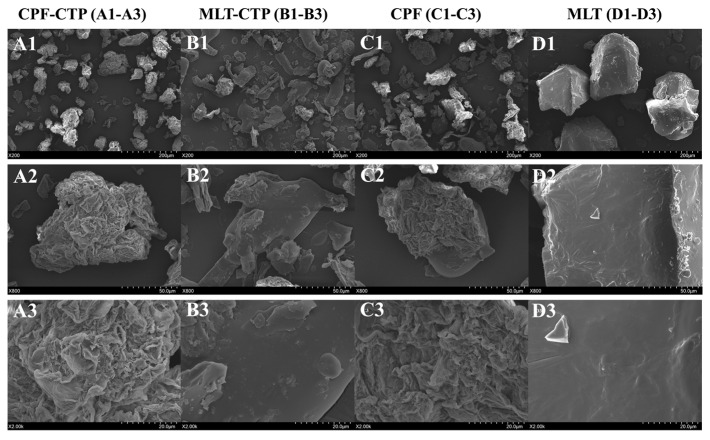
SEM micrographs of CPF-CTP (**A1**–**A3**), MLT-CTP (**B1**–**B3**), CPF (**C1**–**C3**), and MLT (**D1**–**D3**) at magnifications of 200×, 800×, and 2000×.

Scanning electron microscopy (SEM) was used to investigate the surface morphology and microstructural characteristics of citrus peel fibre (CPF), maltitol (MLT), and the corresponding chewable tablet formulations (Figure 4). The surface topology, morphology of particles, and structural changes in pharmaceutical materials are commonly studied with the help of SEM, which illuminates the relationship between microstructure and functional performance [22]. SEM micrographs of CPF (C1–C3) revealed a very irregular, fibrous, and porous structure with an apparent inter-fibrillar gap and loosely packed particles with rough surfaces. The structure appeared as a network of cellulose fibres with significant surface variations and magnifications of higher order (2000×). This structure is typical of lignocellulosic materials that consist of porous cellulose microfibrils within a hemicellulose and pectin matrix [25]. Due to their increased capillary action and surface area, these structural features have been known to enhance hydration properties, particularly water-holding capacity (WHC) and swelling capacity (SC). The SEM images of MLT (D1–D13) indicated smooth, dense, and crystalline morphology with clear angular particles and minimal surface roughness. Flat surfaces and the absence of pores indicate the existence of a highly organised structure and limited water-absorption capacity. This observation explains why the values of WHC and SC of MLT-based formulations were observed to be significantly lower before [37]. The morphology of the formulation CPF-CTP (A13) was altered with respect to raw CPF, with partially compacted and aggregated components. The fibrous network observed in CPF appeared squeezed, resulting in reduced pore size and increased contact between the particles. Most importantly, though, the structure still had surface defects and micro-scale porosity, implying that the structural design of CPF did not completely deteriorate during compression. This partial preservation of porosity is necessary because it allows water to enter and affects the disintegration behaviour and hydration capacity of the tablets reported.

The SEM images of MLT-CTP (B1–B3) contained particles that were flattened, densely packed, and had a more compact and fused structure. The morphology suggested stronger particle fusion and less surface roughness, as it was significantly less porous and more uniform than CPF-CTP. This dense structure is likely to limit water penetration and is related to a reduced level of functional performance regarding swelling and hydration. A comparison of all samples shows that the morphology in systems based on MLT is tightly packed, but the morphology in systems based on CPF is hierarchical porous. Since the SEM analysis reveals surface holes, fissures, and aggregation behaviour, which have a direct impact on product performance, it is particularly effective in detecting such structural differences. Also, the compressive action during the formulation of the tablet is indicated by the shift from a loose fibrous form (CPF) to a partial compact matrix (CPF-CTP). This structural change is confirmed to have no impact on the maintenance of micro-porosity, which ensures that the functional properties of CPF remain intact, justifying its suitability as a multifunctional excipient [31]. One way or another, SEM analysis reveals the morphological changes that occur in the course of formulation and provides good visual evidence of the structural differences between CPF and MLT. The results confirm that CPF maintains a balance between porosity and structural integrity, as required to provide chewable tablets with the needed hydration properties and mechanical stability.

### 3.3. Physicochemical Properties Analysis

#### 3.3.1. Water-Holding Capacity (WHC) and Oil-Holding Capacity (OHC) Analysis

The hydration and binding qualities of the four fibre formulations, namely CPT, CPT-CTP, MLT, and MLT-CTP, were determined by determining their water-holding capacity (WHC) and oil-holding capacity (OHC) (Table 7). Out of these samples, CPF-CTP showed significantly improved functional performance, as it was shown to be markedly more effective in all three parameters [22]. The increase is probably due to the synergistic action of chemical pretreatment and calcium carbonate-aided high-pressure homogenization. These mechanisms are generally claimed to destabilise intra-fibre hydrogen bonding, destabilise crystalline domains, and produce a freer, porous microstructure [8]. This type of structural rearrangement significantly enhances the surface area available and exposes more hydrophilic and lipophilic moieties, resulting in enhanced affinity of the fibre to water, oil, and swelling behaviour.

#### 3.3.2. Particle Size Distribution (PSD) Analysis

**Figure 5:**
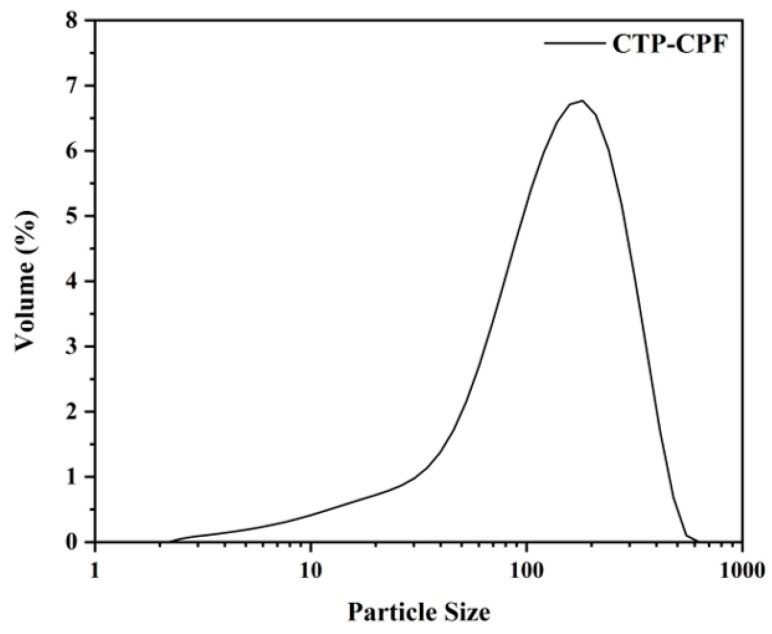
Particle size distribution curve.

The particle size distribution (PSD) analysis of the citrus peel fibre (CPF)-based formulation was used to determine the size properties of the powder mix and its homogeneity (Figure 5). Most particles were grouped in the middle particle size range (100~200 µm), as was observed by the unimodal pattern distribution of the PSD curve and the dominating peak [25]. Significant percentile properties, including D10, D50, and D90, were determined in order to measure the distribution further. It was determined that the values were roughly 45 µm (D10), 140 µm (D50), and 520 µm (D90). As D10 is the fraction of fine particles, D50 is the median particle size, and D90 is the coarse particle fraction, these parameters provide a comprehensive description of the particle size distribution [11]. Such percentile-based metrics are often used to characterise particulate systems and assess their appropriateness in pharmaceutical applications. The formulation is majorly composed of medium-sized particles, as reflected in the calculated D50 of 140 µm, indicating that half of the particles are smaller than this. Owing to its balance between flowability and compressibility, this particle size range is believed to be the best when it comes to direct compression processes. The larger particles (D90 = 520 µm) are used to achieve better packing and mechanical strength, and the smaller particles (D10 = 45 µm) are used to achieve greater surface area and greater water interaction and swelling behaviour.

To further determine the breadth of the distribution, the span value (D90–D10)/D50 was calculated, and it was approximated to be 3.39. This implies the presence of both fine and coarse particles in the system, which has a relatively broad distribution of particle sizes. These distributions occur in fibrous and natural materials, in which variability in the shape of particles causes structural variability. With everything put aside, the PSD analysis confirms that the CPF-based formulation possesses a suitable particle size distribution to be used in producing chewable tablets. The balance between homogeneity in particle size and breadth in their distribution supports the use of it in direct compression-based formulations, as it ensures ideal flow behaviour, compressibility, and functionality [24].

### 3.4. Evaluation of CPF-Enriched Chewable Tablets

#### 3.4.1. Physical Appearance and Structural Integrity

**Figure 6:**
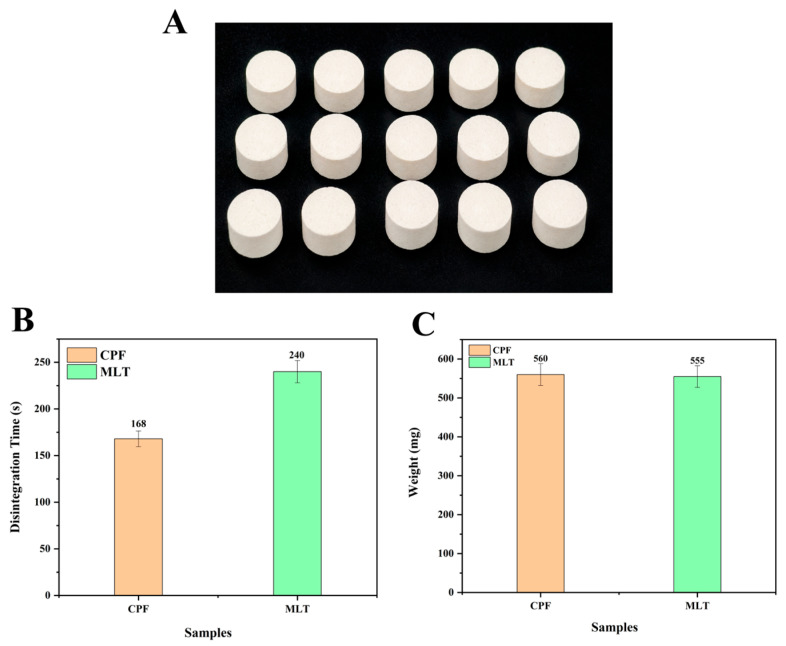
(**A**–**C**). Physical appearance (**A**), disintegration time (**B**), and weight variation (**C**) of CPF chewable tablets.

The chewable tablets were physically examined in terms of their physical characteristics, such as shape, surface texture, colour, and general appearance (Table 8 and Figure 6A–C). The tablets were found to have a circular and planar shape, with a smooth and uniform surface. Tablet shape is a very important quality factor in pharmaceutical formulation since it influences patient acceptability, ease of handling, and manufacturing capability [30]. The two most common shapes are round tablets and flat tablets because compressing them is easy, and the compressive force is evenly spread in the fabrication of the tablet. The round and flat shapes were appropriate in the formulation of chewable tablets, as these shapes provide not only comfort in chewing but also a consistent mouthfeel. Flat surfaces enhance contact area during the mastication process, making the tablet matrix easily disintegrable, which is particularly beneficial in chewable dosage forms. Furthermore, flat tablets are less prone to a mechanical stress concentration compared to irregular shapes, which increases structural integrity when handling and packaging.

Tablet design has a tremendous impact on patient adherence and product acceptability [26]. The studies have shown that round tablets are mostly preferred due to their ease of manufacture and the familiarity of the patients with them; however, flat shapes are usually used in chewable and orally disintegrating forms in order to make tablets easier to swallow and administer. In addition, as per the standard of pharmaceuticals, tablets are mostly made round, flat, or biconvex, depending on formulation requirements and usage. The shape uniformity observed in this experiment indicates uniform filling of the die and sufficient compression when making the tablets, the direct result of good flow characteristics of the powder mixture identified in the pre-formulation experiments [18]. The physical attributes observed confirm the effective formulation and compression of the chewable tablets.

#### 3.4.2. Weight Variation Analysis

The weight difference in the prepared chewable tablets was established to be 560 ± 10 mg (Table 9 and Figure 6C), which represents a steady distribution of the powder mixture during the compression of the tablets. The weight variation test is an important quality control tool that is used to test the consistency of dosage units, ensuring that all the tablets have the same amount of active and inert substances. Through this test, the consistency of filling the die and the flow properties of the powder blend during compression can be determined indirectly. According to the pharmacopoeial rules, tablets with an average weight of more than 250 mg should have a tolerable deviation limit of 5 percent; no more than two tablets may exceed this percentage deviation, and no single tablet may deviate by more than twice that limit. The difference noted in this experiment is acceptable, as it shows that the formulation meets the quality requirement of weight uniformity. The low level of standard deviation observed in the weight of the tablets suggests that the powder blend was able to achieve high flowability and uniform filling of the die. This finding supports the previously obtained micromeritic parameters, including angle of repose (26.62°), bulk density (0.58 g/cm^3^), and Hausner ratio (1.22), which are indicative of desirable flow properties [8]. Therefore, a smooth flow ensures the consistent filling of the die chamber, directly affecting the similar weight of tablets.

Additionally, weight changes are closely interconnected to the hardness and mechanical integrity of the tablets. Equal distribution of compressive force due to the uniform weight of tablets ensures that they are of uniform hardness, and there is minimal variation in the performance of the tablets. Poor weight differences may lead to inconsistencies in doses, undermining therapeutic effectiveness and patient safety, therefore making it an essential quality feature in tablet formulation. The results of this study show that the optimised formulation and compression process were effective in producing tablets with a consistent weight. This indicates sufficient incorporation of excipients, good powder flow, and stable compression parameters. The findings also confirm that the formulation is viable for large-scale manufacturing, where uniform weight is vital in meeting regulatory requirements and ensuring the quality of the products.

#### 3.4.3. Thickness and Diameter Examination

**Figure 7:**
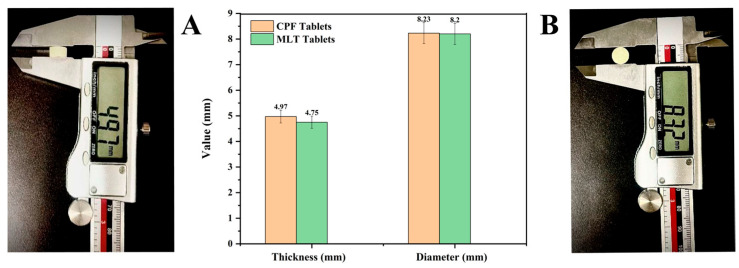
(**A**) Thickness and (**B**) diameter of CPF chewable tablets.

The formulated chewable tablets were of homogeneous dimensions and uniform compression, as the thickness of the final tablets was 4.97± 0.60 mm (Table 9 and Figure 7A,B). The thickness of tablets is the distance between the upper and lower surfaces of a tablet and is an important physical characteristic of the quality of pharmaceuticals, as it directly influences packaging, mechanical properties, and release characteristics of drugs. The difference in the thickness measurements is low, which means that the powder mixture exhibited uniform die filling and die compression behaviour [19]. The thickness is largely predetermined by factors such as compression force, die fill volume, and material properties. Increased compression force tends to produce thinner and harder tablets, and decreased compression force produces thicker and softer tablets. The uniform thickness obtained in this study indicates that the compression parameters were well adjusted, resulting in the production of uniform tablets. In addition, the dissolving and disintegrating properties of the dosage form are greatly affected by tablet thickness. Thermally resistant tablets tend to disintegrate and dissolve more slowly, whereas thinner tablets can disintegrate faster. Therefore, maintaining thickness in a controlled range is essential in maintaining a consistent drug release and therapeutic response. The values of thickness are in line with the earlier obtained micromeritic values of the powder blend [21]. The excellent flowability, demonstrated by the low angle of repose (26.62°), and the desirable compressibility, indicated by Carr index (8.9) and Hausner ratio (1.22), enabled homogeneous die filling and homogeneous compaction, which led to homogeneous tablet thickness. This demonstrates a strong association between pre-formulation characteristics and final tablet characteristics. Moreover, consistent thickness is a requisite of effective packaging, especially in blister packaging and automated counting processes, where variability in tablet size may lead to packaging defects or equipment malfunctions. Thus, the homogeneity of thickness confirms that the formulated product meets important quality control criteria for pharmaceutical tablets production [25]. The thickness analysis shows that the developed chewable tablets have consistent dimensional properties, which implies a high-quality formulation design, optimal compression parameters, and good powder flow properties.

#### 3.4.4. Disintegration Analysis

The disintegration time of the prepared chewable tablets was established to be 168 s with a deviation of 15 s, which indicates that there is adequate degradation of the tablet matrix under simulated physiological conditions (Table 9 and Figure 6B). The process of disintegration is an important quality parameter of tablet preparations since it refers to the time needed by a tablet to break into smaller fragments, which are necessary to break down the medication to be absorbed [22]. Although disintegration testing is not always mandatory to evaluate chewable tablets, since they are designed to be disintegrated by chewing, it is a major criterion to evaluate the inherent disintegration properties of the formulation. The disintegration time of approximately 168 s (roughly 2.8 min) indicates that the tablets can effectively disintegrate after being mashed, which consequently releases the contents within them. Compared to the pharmacopoeia requirements of traditional uncoated tablets, which typically require disintegration within 15 min, the developed product has a significantly quicker disintegration profile. The rapid breakdown is beneficial, as it allows for faster access to active substances and enhances the effectiveness of the dose form. Formulation composition and compression properties greatly influence the observed disintegration behaviour. The moderate hardness (2.53 ± 0.80 kg) obtained in this experiment provides sufficient integrity to the tablet while ensuring it disintegrates quickly when subjected to a wet environment. Lower hardness is likely to increase the disintegration rate due to increased porosity and decreased bonding between particles [28].

Furthermore, hydrophilic excipients, such as microcrystalline cellulose and oligosaccharides, promote absorption and swelling in water, thus increasing the disintegration of tablets. The dietary fibre, citrus peel fibre, has the potential to increase water absorption and matrix disruption, and thereby increase the process of breaking down. The results of the disintegration are in accordance with the previously defined micromeritic properties, such as excellent flowability (angle of repose 26.62°) and good compressibility (Carr index 8.9% and Hausner ratio 1.22), which enabled a homogeneous structure of the pill and recommended porosity. All these variables helped in the successful permeation of the dissolution media and rapid breakage of the tablets. The disintegration study shows that the developed chewable tablets have rapid and efficient degradation characteristics, which enable the components to be released with ease, supporting their suitability for chewable tablet formulations.

## 4. Conclusions

This study successfully developed fibre-enriched chewable tablets via direct compression using pomelo (*Citrus maxima*) peel fibre as a functional ingredient and structural matrix. Compared with the maltitol-based control, the fibre-enriched formulation exhibited improved powder flowability, satisfactory micromeritic behaviour, and compressibility, supporting uniform tablet production without adversely affecting tablet manufacturability. The incorporation of pomelo peel fibre significantly enhanced water-holding and oil-holding capacities, which may be associated with its porous and fibrous microstructure, as confirmed by scanning electron microscopy. Structural analyses further confirmed the presence of characteristic lignocellulosic functional groups, reduced crystallinity, and irregular porous morphology within the CPF matrix. In contrast to the crystalline structure of the control system, the fibre-based formulation showed a semi-crystalline organisation, which was associated with modified hydration-related properties. The resulting tablets exhibited acceptable physicochemical properties, including uniform weight, consistent dimensions, structural integrity, and reproducible disintegration characteristics under direct compression conditions. Overall, pomelo peel fibre shows potential as a technologically functional ingredient in chewable tablet systems while also supporting the valorization of citrus processing by-products in functional food development. However, the findings of the present study are limited to the physicochemical, structural, and technological characterisation of the developed formulations. Therefore, future studies should focus on advanced formulation optimisation, storage stability, simulated gastrointestinal digestion, bioaccessibility assessment, and in vivo functionality to further clarify the potential practical applications of CPF-enriched chewable tablet systems.

## Figures and Tables

**Table 1:** Preparation of tablet excipient formulations with pomelo peel fibre (CPF) and maltitol (MLT) control group.

Ingredients	CPF Tablet (g)	MLT Control Tablet (g)
Citrus Peel Fibre (CPF)	4.50	-
Maltitol (MLT)	-	4.50
Oligosaccharide	2.50	2.50
Orange Powder	1.0	1.0
Microcrystalline Cellulose (MCC)	1.00	1.00
Stevia (Sweetener)	0.50	0.50
Citric Acid	0.30	0.30
Magnesium Stearate	0.20	0.20
Total Weight	10.0 g	10.0 g

**Table 2:** Experimental measurements of height, radius, and calculated angle of repose (°) of CPF and MLT.

Formulation	Height (cm)	Radius (cm)	Angle of Repose (°) CPF	Height (cm)	Radius (cm)	Angle of Repose (°) MLT
1	3.00	6.50	24.77	3.80	5.90	32.94
2	3.70	6.00	31.71	4.10	5.80	35.44
3	2.90	6.40	24.66	3.60	6.0	31.0
Mean ± SD	—	—	26.62 ± 3.53	—	—	33.11 ± 2.25

**Table 3:** Bulk density (g/cm^3^) and tapped density (g/cm^3^) of the powder blend.

**Formulation**	**Mass (g)** **CPF**	**Volume (cm^3^)** **CPF**	**Bulk Density (g/cm^3^) CPF**	**Mass (g)** **MLT**	**Volume (cm^3^)** **MLT**	**Bulk Density (g/cm^3^) MLT**
1	10	17.5	0.57	10	15.5	0.65
2	10	17.0	0.59	10	15.2	0.66
3	10	17.2	0.58	10	15.4	0.65
Mean ± SD	—	—	0.58 ± 0.02	—	—	0.65 ± 0.01
**Formulation**	**Mass (g)** **CPF**	**Tapped Volume (mL) CPF**	**Tapped Density (g/cm^3^) CPF**	**Mass (g)** **MLT**	**Tapped Volume (mL) MLT**	**Tapped Density (g/cm^3^) MLT**
1	10	14.5	0.69	10	12.8	0.78
2	10	14.0	0.71	10	12.5	0.80
3	10	13.5	0.70	10	12.3	0.81
Mean ± SD	—	—	0.71 ± 0.03	—	—	0.79 ± 0.01

**Table 4:** The calculated Hausner ratio of the powder blend indicates flowability characteristics.

Parameter	CPF Value	MLT Value
Bulk Density (g/cm^3^)	0.58	0.65
Tapped Density (g/cm^3^)	0.71	0.79
Hausner Ratio	1.22	1.22

**Table 5:** Carr’s index (%) of the powder.

Formulation	CPF (Carr’s Index %)	MLT (Carr’s Index %)
1	7.2	17.7
2	10.5	18.9
3	9.0	17.5
Mean ± SD	8.9 ± 2.11	18.0 ± 0.75

**Table 6:** Comparative studies of powder formulations (CPF vs. maltitol control).

Parameter	CPF (Mean ± SD)	MLT (Mean ± SD)	Standard Range	Interpretation (CPF vs. MLT)	Flow Character
Angle of Repose (°)	26.62 ± 3.53	33.11 ± 2.25	25–30°	CPF: Excellent flow MLT: Good flow	CPF: Excellent MLT: Good
Bulk Density (g/cm^3^)	0.58 ± 0.02	0.65 ± 0.01	—	CPF: Moderate packing MLT: Denser packing	—
Tapped Density (g/cm^3^)	0.71 ± 0.03	0.79 ± 0.01	—	CPF: Moderate densification MLT: Higher densification	—
Hausner Ratio	1.22	1.22	1.19–1.25	Both: Fair–Good flow	Good
Carr’s Index (%)	8.9 ± 2.11	18.0 ± 0.75	≤10% (Excellent)	CPF: Excellent compressibility	CPF: Excellent MLT: Good
Overall Flow Property	—	—	—	—	CPF: Excellent MLT: Good

**Table 7:** Water-holding capacity (WHC) and oil-holding capacity (OHC) of powder formulations.

Sample	WHC (g/g)	OHC (g/g)
CPF-CTP	9.84 ± 2.49	2.97 ± 0.07
CPF	5.99 ± 0.35	2.58 ± 0.06
MLT	0.82 ± 0.13	1.91 ± 0.20
MLT-CTP	1.08 ± 0.04	2.10 ± 0.07

**Table 8:** Physical appearance evaluation of chewable tablets.

Parameter	Observation
Shape	Round and flat
Surface	Smooth
Colour	Light orange
Odour	Pleasant citrus
Texture	Uniform

**Table 9:** Thickness, diameter, weight, and disintegration time of CPF and MLT chewable tablets.

Parameters	CPF Tablets (Mean ± SD)	MLT Tablets (Mean ± SD)
Thickness (mm)	4.97 ± 0.60 ^a^	4.75 ± 0.50 ^a^
Diameter (mm)	8.23 ± 0.13 ^a^	8.20 ± 0.10 ^a^
Weight (mg)	560 ± 10 ^a^	555 ± 8 ^a^
Disintegration Time (s)	168 ± 18 ^b^	215 ± 35 ^a^

Values are expressed as mean ± standard deviation (*n* = 3). Significant differences were observed between CPF and maltitol formulations (*p* < 0.05). Different superscript letters within the same row indicate significant differences between formulations.

## Data Availability

The data presented in this study are available on request from the corresponding author.
